# Development of a new approach to diagnosis of the early fluorosis forms by means of FTIR and Raman microspectroscopy

**DOI:** 10.1038/s41598-020-78078-8

**Published:** 2020-12-01

**Authors:** Pavel Seredin, Dmitry Goloshchapov, Yuri Ippolitov, Jitraporn Vongsvivut

**Affiliations:** 1 Department of Solid State Physics and Nanostructures, Voronezh State University, University Sq. 1, Voronezh, Russia 394018; 2 Ural Federal University, 19 Mira Street, Ekaterinburg, Russia 620002; 3 Department of Pediatric Dentistry With Orthodontia, Voronezh State Medical University, Studentcheskaya St. 11, Voronezh, Russia 394006; 4 Australian Synchrotron (Synchrotron Light Source Australia Pty LTD), 800 Blackburn Rd, Clayton, VIC 3168 Australia

**Keywords:** Diagnostic markers, Infrared spectroscopy, Raman spectroscopy

## Abstract

This study is aimed at investigating the features of mineralization of the enamel apatite at initial stages of fluorosis development. Samples of teeth with intact and fluorotic enamel in an early stage of the disease development (Thylstrup–Fejerskov Index = 1–3) were studied by Raman scattering and FTIR using Infrared Microspectroscopy beamline at Australian Synchrotron equipment. Based on the data obtained by optical microspectroscopy and calculation of the coefficient R [A-type/B-type], which represents the ratio of carbonation fraction of CO_3_^2−^, replacing phosphate or hydroxyl radicals in the enamel apatite lattice, the features of mineralization of enamel apatite in the initial stages of development of the pathology caused by an increased content of fluorine in the oral cavity were established. Statistical analysis of the data showed significant differences in the mean values of R [A-type/B-type] ratio between the control and experimental groups for surface layers (*p* < 0.01). The data obtained are potentially significant as benchmarks in the development of a new approach to preventive diagnostics of the development of initial and clinically unregistered stages of human teeth fluorosis, as well as personalized control of the use of fluoride-containing caries-preventive agents.

## Introduction

It has been established that caries prevalence is associated with the intake of fluorine compounds^1,2^, that is, the less fluoride, the higher is caries frequency^3,4^. The program to introduce water fluorination in several European countries has led to a reduction in the number of carious pathologies in the population^1^. At the same time, it was shown that fluorination is not a panacea^1,2^, especially under excessive fluoride intake in cases of medium severity leading to osteosclerosis and osteoporosis^2^. According to WHO, redundant fluoride in drinking water, as well as the irrational use of fluoride toothpaste, leads to the development of fluorosis^2,5,6^. The excess of fluorine affects millions of people globally, although it is most often manifested in light and medium-light forms^2,7^.

Enamel fluorination is one of the methods to combat initial caries^5,8^, stabilising the inorganic part of the dental matrix, nanocrystalline defective calcium carbonate substituted hydroxyapatite (CHAP), which in case of biogenic apatite can be described by the structural formula Ca_8.8_Mg_0.1_(PO_4_)_4.9_(HPO_4_)_0.6_(CO_3_)_0.5_(OH)_0.9_^9^ or more precisely (Ca)_5.x_(Mg)_q_(Na)_u_(HPO_4_)_v_(CO_3_)_w_(PO_4_)_3.y_(OH,F)_x_z_ where x, q, u, v, w, y, and z are the stoichiometry coefficients^10^. Fluorine ions replace hydroxyl groups and vacancies in the apatite, forming a more stable crystal lattice, calcium fluorapatite (CFA)^8,10–13^, which is less susceptible to caries. However, the formation of FA, given its physical and chemical properties different from those ones of native apatite, leads to a disrupted mineralization and transport functions of enamel^5,8,14^. Prolonged exposure of teeth to substances containing high levels of fluoride can lead to the formation of cracks and chips due to the formation of calcium fluoride (CaF_2_) enamel in the subsurface layers^5,12,14^. Consequently, many individuals, due to excess exposure to toothpaste and other fluorine-containing components, have an increased fluorosis^5–7^. The indicators of prevalence and severity of this pathology in developed countries have increased not only in time but by orders of magnitude.

As a result of fluoride accumulation, fluorosis does not manifest clinically until the colour and morphology of the teeth change, which can be observed during the visual examination of the patient^14–16^. This requires the development of a diagnostic technique for the enamel condition to register changes in the enamel matrix during the development of fluorosis^17–19^. Early diagnosis of dental diseases using precision monitoring methods is a key paradigm for ensuring public^15,20–24^, hence the diagnosis of this disease at different levels of development is a priority scientific direction in therapeutic dentistry in the developed countries.

Since the condition of human teeth is determined by changes occurring in the phase composition of the tissue at the micro and nano level, the use of spectroscopic methods of molecular identification is the most promising and sensitive tool to precisely assess such changes in the dental enamel^24–26^. Raman spectroscopy is most often used for diagnostics of dental diseases as a non-destructive method of analysis, which allows to obtain direct data regarding the local atomic structure, chemical and molecular composition of biological objects^18,20,24,27–29^. Also, the hard tissue of a human tooth can be investigated by infrared spectroscopy methods^30–34^, which due to minimal external influences, has not been modified by the method^35^.

At the same time using the IR-microspectroscopy with synchrotron radiation as a source allows one to analyze biological specimens (human tooth tissues) and pathology processes in them with a greater lateral resolution during data acquisition and high signal-to-noise ratio without the long-term accumulation of the desired signal that is important for the study of biological specimens^36–38^. The use of infrared microspectroscopy with synchrotron radiation makes it possible to clarify the molecular composition and study local changes in the mineral-organic matrix of dental enamel from the areas less than 10 µm^2^^39^. Moreover, in any case the applicability of synchrotron IR-microspectroscopy to the dental tissue analysis is more convenient than Raman microspectroscopy, even though the Raman microscopy is also an excellent approach to vibrational spectroscopy, with an equivalent, or often a better, spatial resolution than synchrotron infrared microscopy^31,33,37,40^.

It should be noted that the correlation between the data obtained by Raman methods and infrared (IR) microspectroscopy for the samples of dental hard tissue affected by fluorosis, taking into account the thickness of the enamel, was not made. However, separate studies of the molecular structure of teeth with fluorosis by methods of optical microspectroscopy have been performed^14–16,18,41^. Therefore, this study is aimed to investigate the peculiarities of mineralization of enamel apatite in the initial stages of fluorosis using Raman and IR microspectroscopy to determine the potential of the obtained data for the development of a new method of diagnostics of early forms of fluorosis.

## Results

### Raman microspectroscopy

**Figure 1:**
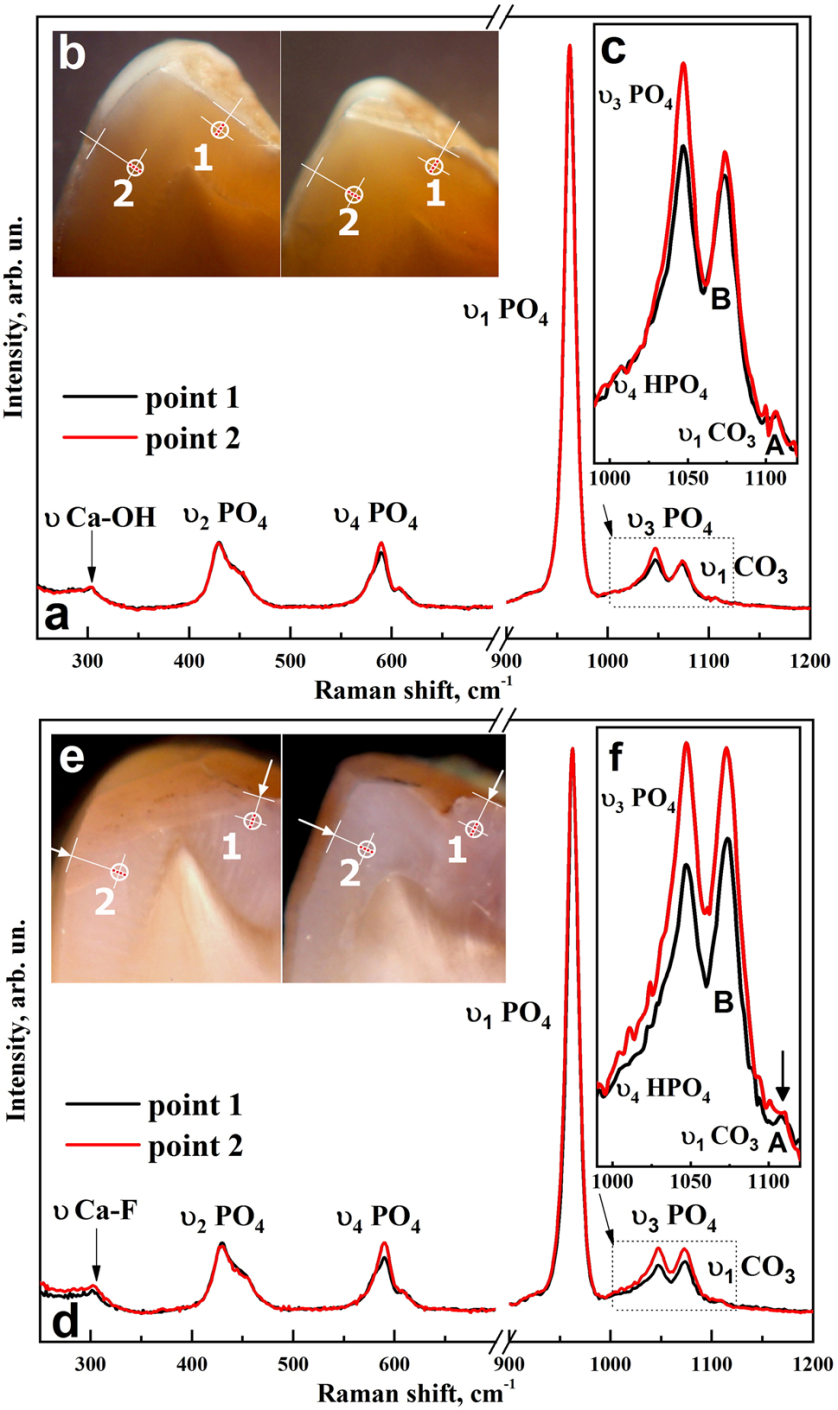
Raman spectra of the: (**a**) intact tooth enamel with the points (**b**) where they were collected from micro-areas of the enamel of typical healthy teeth samples and direction of their scanning and (**c**) Raman vibrations of CO_3_^2−^ in A- and B-type positions in CHAP spectra of the control group samples; Raman spectra of the (**d**) enamel of a tooth with fluorosis, with the points (**e**) where they were collected from micro-areas of the enamel of typical specimens of teeth with fluorosis and the direction in which they are scanned, and (**f**) vibrations of CO_3_^2−^ at A- and B-type positions in the CHAP spectra of the experimental group specimens.

Two macro-areas of enamel were chosen in the cuspal enamel region for each specimen. The first micro-area of interest was arranged near the surface of enamel where according to Gerth et al.^14^ and Campillo et al.^17^, the most prominent changes in Raman scattering are observed associated the development of fluorosis connected with the formation of CaF_2_. Second micro-area was arranged at the half-depth of the investigated enamel layer where in accordance with Gerth et al.^14^, Tsuda et al.^16^ and Campillo et al.^17^ the changes in vibrational characteristics of enamel apatite are possible due to inclusion of fluorine ions into its crystal lattice. Next, micro-areas were chosen within the macro-regions of enamel (fluorotic/intact) in order to measure Raman signal. The choice of micro-areas was determined by the absence of morphological, erosive, surface changes in the enamel and by fulfillment of geometrical conditions for spectral data acquisition as well as by signal-to-noise ratio. Therefore, for the sake of clarity, Fig. 1a and d present the Raman spectra averaged in groups obtained from micro-areas of healthy and fluorotic teeth samples. Figure 1b and e show the micro-areas of the enamel in typical samples of healthy and fluorotic teeth selected for examination, as well as the direction of scanning of Raman microspectroscopy.

The analysis of active vibrations in Raman spectra was performed basing on several sources where hard dental tissue in normal and pathology was investigated by Raman scattering^14,15,17,20,27,30,35,42–44^. According to the literature data, the spectra include the most intense vibrations associated with ν_1_ and ν_3_ PO_4_^3−^ CHAP localised at 962.6 cm^−1^ and 1006.2, 1030, 1047.4 cm^−1^ respectively^14,20,34,35,45–48^. Besides, in all Raman spectra, there are maxima associated with the inclusion of carbonate ion CO_3_^2−^ in the apatite lattice (Fig. 1c and f). The peak located at 1073 cm^−1^ is the CO_3_^2−^ vibration, which replaced PO_4_^3−^ (B-substitution type) in the CHAP lattice^48^. The low-intensity vibrations localised at 1106 cm^−1^ is associated with the inclusion of CO_3_^2−^ into the OH- (A-substitution type) group position in the HAP lattice. The results of preliminary data analysis showed that Raman spectra of intact and fluorotic enamels contained a similar set of vibrational modes (see Table 1), differing in intensity at the different scanning points.

**Table 1:** Active vibrations in Raman and IR spectra and their molecular groups.

Vibrations groups	Raman, cm^−1^	IR, cm^−1^	Type of vibration	Refs.
Ca_II_–OH	305		Trans. HAP	^16^
Ca_II_–F	311		Trans. FAP	^42^
υ_2_ PO_4_	431		O–P–O bend	^15,17,20,28,34,42–47,49^
υ_2_ PO_4_	447		O–P–O bend	^15,17,20,34,42–44,46,47,49^
υ_2_ PO_4_	454		O–P–O bend	^15,17,20,34,42–44,46,47,49^
υ_4_ PO_4_	579		O–P–O bend	^15,17,20,34,42–44,46,47,49^
υ_4_ PO_4_	590		O–P–O bend	^15,17,20,34,42–44,46,47,49^
υ_4_ PO_4_	607		O–P–O bend	^17,34,42,43^
υ_4_ PO_4_	614		O–P–O bend	^17,34,42,43^
υ_1_ CO_3_ A-type		870	C–O	^30,34,40^
υ_1_ CO_3_ B-type		890	C–O	^30,34,40^
υ_1_ PO_4_	962	956.6	P–O str	^15,17,20,28,30,34,40,42–47,49,50^
HPO_4_	1005		P–O sym. str	^44,49,51,52^
υ_3_ PO_4_	1028			^17,43,44,53,54^
υ_3_ PO_4_	1040	1040	P–O antysym. str	^17,30,40,42–44,53,54^
υ_3_ PO_4_	1047	1048	P–O antysym. str	^17,30,40,42–44,53,54^
υ_3_ PO_4_	1052	1060	P–O antysym. str	^17,30,40,42–44,53,54^
		1090	P–O antysym. str	^17,30,40,42–44,53,54^
υ_1_ CO_3_ B-type υ_1_ CO_3_ AB-type/CH_3_	1070–1072	1401.3 1445	C–O	^30,35,40,45–47,49,54,55^
υ_3_ PO_4_	1076–1077		P–O antysym. str	^17,18,44–46^
υ_1_ CO_3_ A-type	1106	1540	C–O	^30,35,40,45–47,49,54,55^

**Figure 2:**
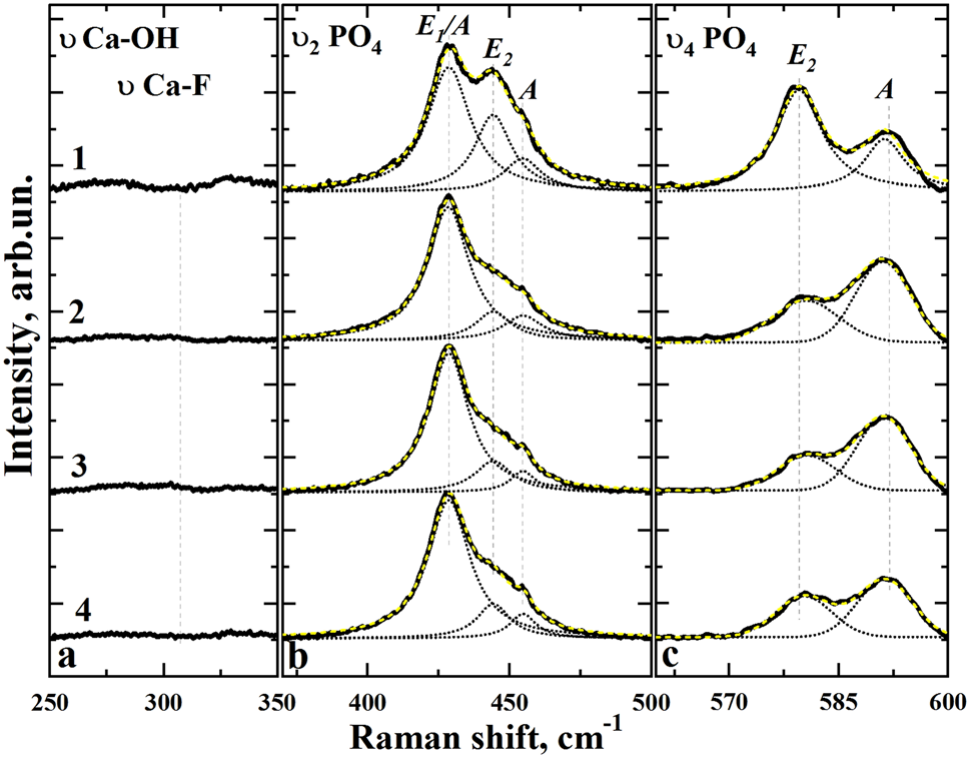
Raman spectra in the vibration area: (**a**) Ca_II_—OH and Ca_II_ -F; (**b**) PO_4_^3−^ ν_2_; (**c**) PO_4_^3−^ ν_4_; collected in micro-areas of enamel samples of the control group in the scanning direction from the enamel surface to dentin in 100 µm increments. 1—surface, 2—depth 100 µm, 3—depth 200 µm, 4—depth 300 µm.

**Figure 3:**
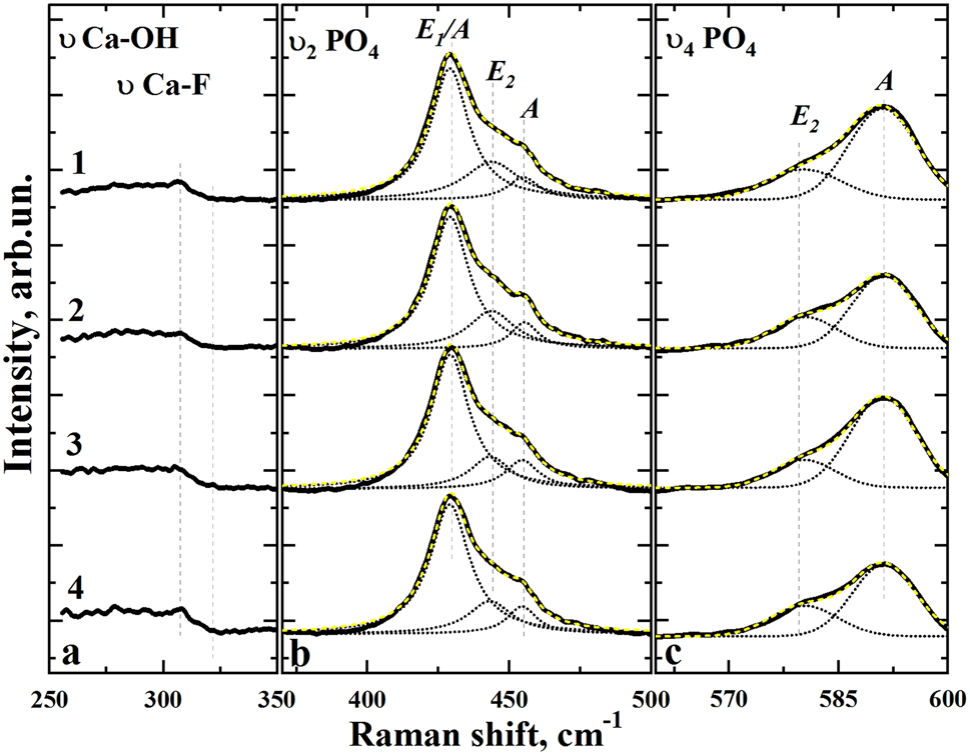
Raman spectra in the vibration region: (**a**) Ca_II_—OH and Ca_II_ -F; (**b**) PO_4_^3−^ ν_2_; (**c**) PO_4_^3−^ ν_4_, collected in micro-areas of enamel samples of the experimental group in the scanning direction from the enamel surface to dentin in 100 µm increments. 1—surface, 2—depth 100 µm, 3—depth 200 µm, 4—depth 300 µm.

More detailed further analysis of the Figs. 2 and 3 shows low-intensity vibrations in the Raman spectra in the ranges of 250–350 cm^−1^, 350–500 cm^−1^ and 560–600 cm^−1^ collected on micro-areas of enamel samples of both groups in the direction of scanning from the enamel surface to dentin in 100 µm steps.

In the range of 400–600 cm^−1^, the phosphate group fluctuations are attributed to the modes PO_4_^3−^ ν_2_ and PO_4_^3−^ ν_4_ CHAP, localised at 428.4 cm^−1^, 448.2 cm^−1^ and 579.6 cm^−1^, 591.6 cm^−1^ respectively. According to the data of Tsuda et al.^43^ and Leroy et al*.*^46^*.* PO_4_^3−^ ν_2_ and PO_4_^3−^ ν_4_ modes observed in the spectra of the enamel apatite consist of the overlapping vibrations (symmetry species) A, E_1_ and E_2_, characteristic for the point group C_6_ (P6_3_/m) of hydroxyapatite, apatite of enamel and fluoroapatite. In order to identify the regularities of changes in the intensity of Raman scattering bands in the spectra of intact and fluorotic enamel we employed decomposition of PO_4_^3−^ ν_2_ and ν_4_ modes into the components.

On the basis of decomposition results for PO_4_^3−^ ν_2_ and ν_4_ modes one can see (Figs. 2 and 3, Table 2), that positions of A, E_1_ and E_2_ bands in the Raman spectra of the intact and fluorotic enamel are not changed (Figs. 2c and 3c). This is in agreement with the known data from the work of G. Leroy et al.^46^, where a comparison of Raman spectra for the enamel apatite and FAp was employed. The main differences in the spectra of intact and fluorotic enamel are observed as in the intensity as in the half-width of A, E_1_ and E_2_ bands.

**Table 2:** Results of decomposition of υ_2_ and υ_4_ PO_4_^3−^ modes into the components in Raman spectra from the enamel surface.

Vibration/symmetry species	Raman region, cm^−1^	Intact teeth				Fluorotic teeth			
		Surface	Depth 100 µm	Depth 200 µm	Depth 300 µm	Surface	Depth 100 µm	Depth 200 µm	Depth 300 µm
		HWHM, cm^−1^							
PO_4_^3−^ ν_2_ E_1_/A	428–431	18.4	17.8	16.9	17.8	16.1	16.3	16.3	16.8
PO_4_^3−^ ν_2_ E_2_	444–447	15.7	15.3	15.9	14.0	23.3	19.6	17.1	18.5
PO_4_^3−^ ν_2_ A	454–455	15.6	16.9	10.7	11.2	13.2	11.6	13.4	12.5
PO_4_^3−^ ν_4_ E_2_	579–580	7.7	10.8	9.4	9.0	12.1	10.4	9.9	10.9
PO_4_^3−^ ν_4_ A	590–592	6.7	8.7	8.7	8.8	10.9	10.1	10.6	10.2

From Figs. 2c and 3c, the vibration of PO_4_^3−^ ν_4_ is characterised by a change in the mode as the intensity of its components is redistributed. The change of intensities of the E_2_ and A components of PO_4_^3−^ ν_4_ vibration is noticeable when comparing spectra obtained from different layers of the intact enamel. Moreover, the different vibration shape of PO_4_^3−^ ν_4_ is observed when comparing spectra obtained from similar micro-enamel layers of intact and fluorotic teeth.

It should be noted that the redistribution of the intensity of E_2_ and A components PO_4_^3−^ ν_4_ in the region of 579–592 cm^−1^ and PO_4_^3−^ ν_2_ in the region of 428–455 cm^−1^ observed when comparing intact and fluorotic enamel samples, may indirectly indicate at the presence of fluorine in the structure, according to previous data by Leroy et al.^46^ and Penel et al*.*^52^. However, according to Tsuda et al.^43^*.* and Leroy et al.^46^, the redistribution of PO_4_^3−^ ν_4_ band symmetry can be also caused by a different orientation of HAP crystals in enamel prisms. In the case of fluorotic enamels such redistribution is not observed within one micro-area (Figs. 2c and 3c), so the contribution of fluorine inclusions in the apatite structure plays a major role in the intensity transformation.

Moreover, it is well known that in the range of 280–311 cm^−1^ of the Raman spectra Ca_II_–OH hydroxyapatite and Ca_II_–F from fluorapatite (FAp)^42^ vibrations are localized. As it follows from our experimental data (see Fig. 3a) the appearance of low-intensive band in the range of 311 cm^−1^ is observed only in the spectra of fluorotic enamel. This fact corroborates the increase of fluorine content in the hard dental tissue of the specimens from the experimental group as compared with the control one.

### Results of IR reflection microspectroscopy

**Figure 4:**
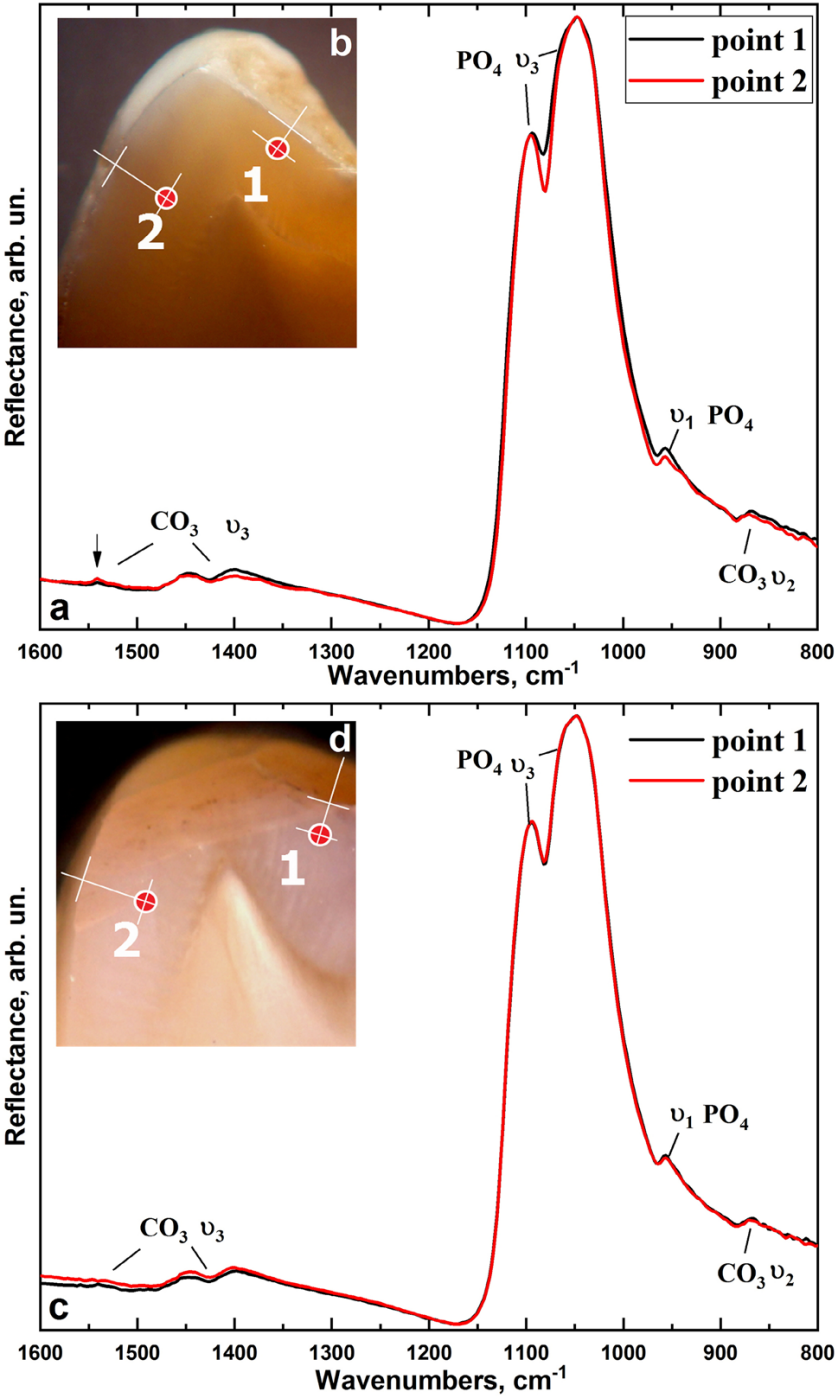
FTIR spectra of the: (**a**) intact tooth enamel with the points (**b**) where they were collected from micro-areas of the enamel of typical healthy teeth samples, and (**c**) enamel of a tooth with fluorosis with the points (**d**) where they were collected from micro-areas of the enamel of typical specimens of teeth with fluorosis.

IR reflection spectra obtained from the same areas of the intact and fluorotic teeth enamel using a synchrotron source are shown in Fig. 4a and c, demonstrating variations associated with the mineral matrix of the enamel (see Table 1). Figure 4b and d show micro-areas of enamel of the typical samples of healthy and fluorotic teeth selected for examination. The maximums in the spectra located at 1094, 1060, 1048, 1040 cm^−1^ are the vibrations of ν_3_ of the radical PO_4_^3−^ and the peak of 956.4 cm^−1^ can be attributed to PO_4_^3−^ ν_1_. In addition, the reflection spectra contain bands associated with CO_3_^2−^, replacing phosphate or hydroxyl radicals (A and B-type substitutions, respectively). Active in the spectra are CO_3_^2−^ ν_3_ in the range of 1540.7 (A-type), 1446.6 (AB-type), 1401.3 (B-type) cm^−1^, and CO_3_^2−^ ν_1_ 879 (A-type—shoulder), 870.6 (B-type) cm^−1^.

**Figure 5:**
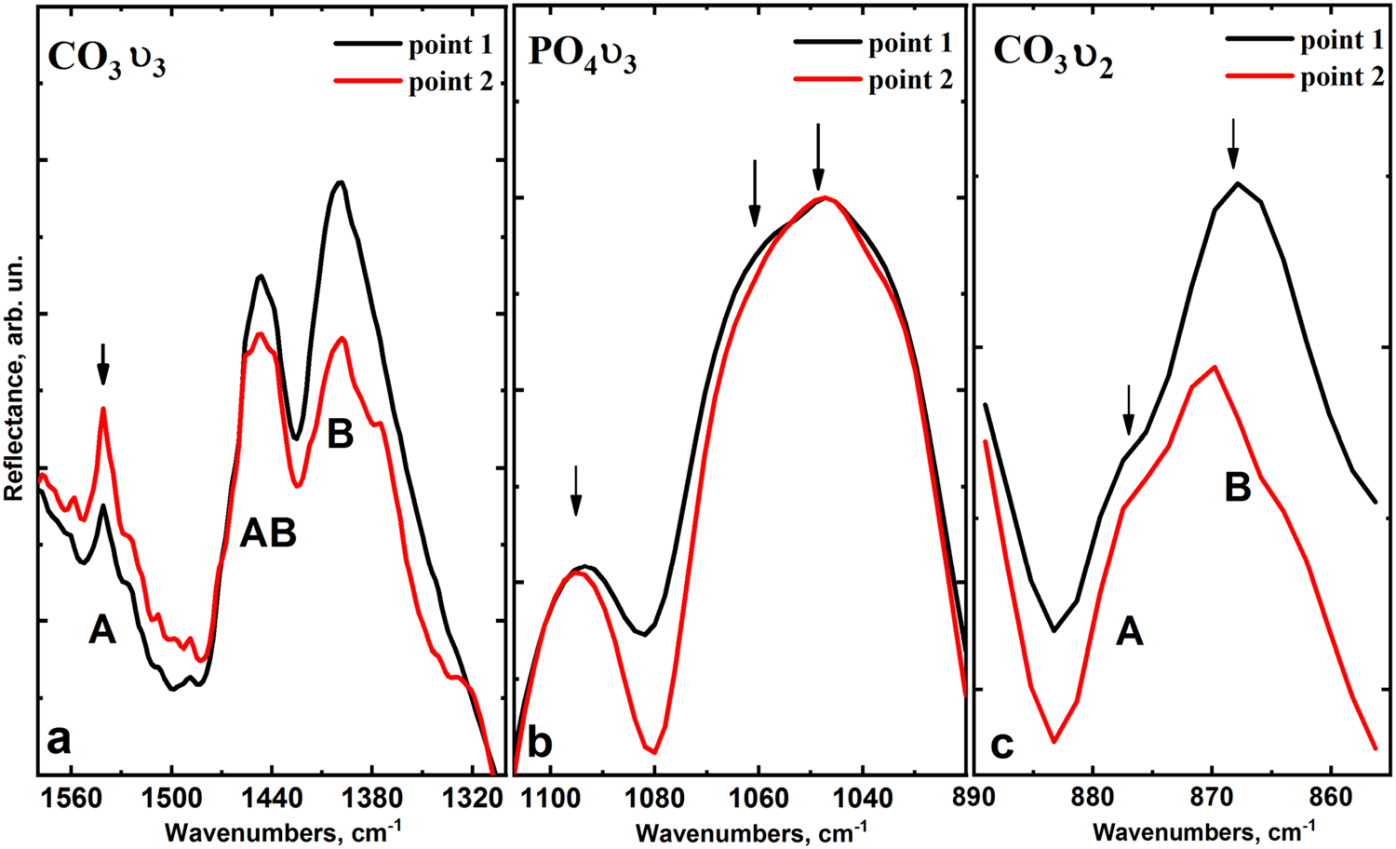
Fluctuations of ν_3_ and ν_2_ of CO_3_^2−^ in positions A- and B-type (**a**,**c**), as well as the maximum of ν_3_ of radical PO_4_^3−^ (**b**) in the IR reflection spectra of control group samples (intact dental tissue).

**Figure 6:**
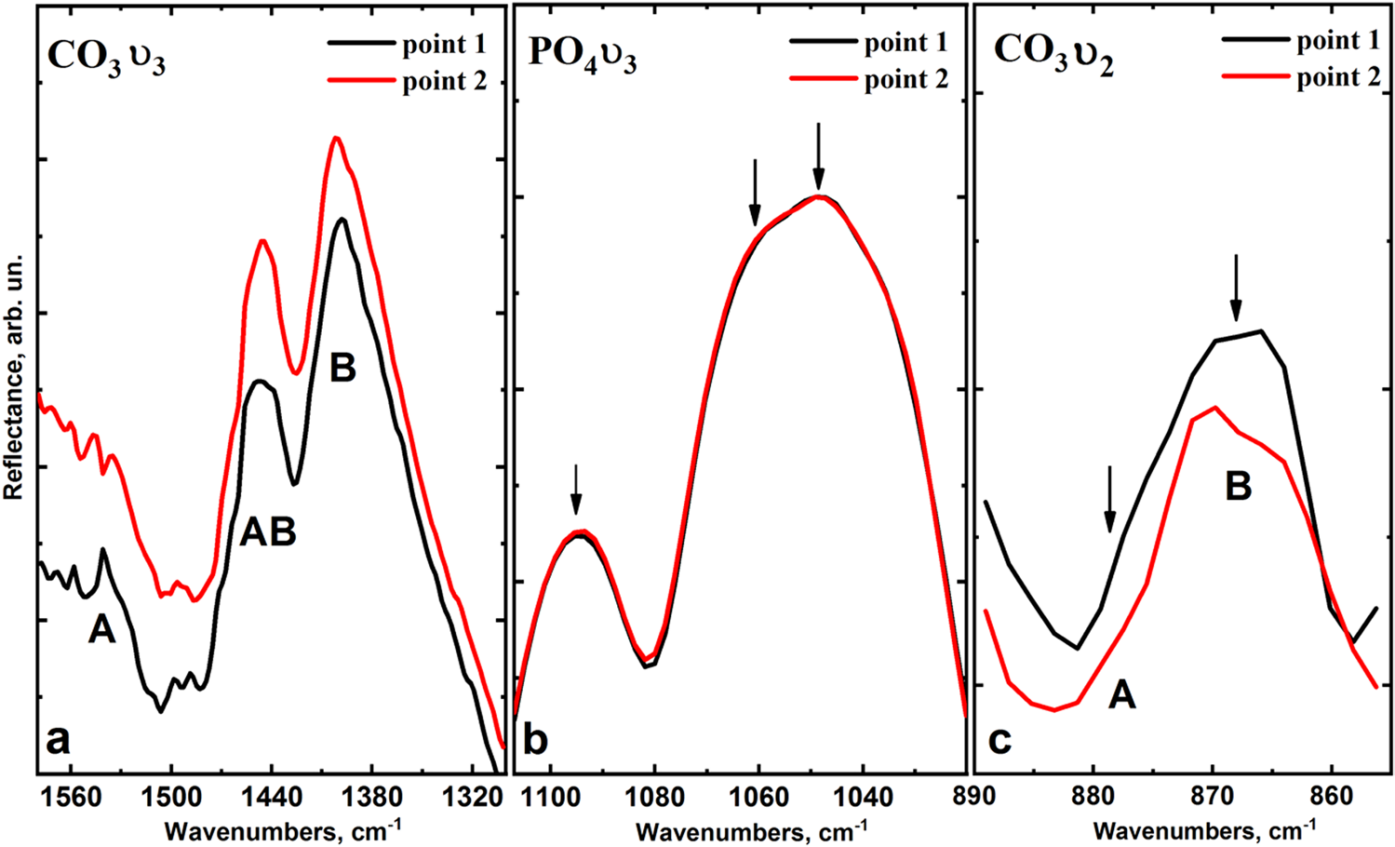
Fluctuations of ν_3_ and ν_2_ CO_3_^2−^ in the positions of A- and B-type (**a**,**c**), as well as the maximum of ν_3_ radical PO_4_^3−^ (**b**) in the IR reflection spectra of the experimental group samples (fluorosis dental tissue).

Figures 5 and 6 show the vibration regions 1580–1320 cm^−1^ and 890–850 cm^−1^ for a more detailed analysis, with the vibrations ν_3_ and ν_2_ of carbon ions CO_3_^2−^ at the positions of A- and B-type as well as the main maximum region ν_3_ of radical PO_4_^3−^ 1000–1100 cm^−1^.

As it is shown in Fig. 5b, in the case of intact teeth, there is a redistribution of components of the high-intensity band ν_3_ of radical PO_4_^3−^ in the infrared reflection spectra from enamel areas located at different depths, which is in agreement with the literature^34^. Similarly, the redistribution of intensity is observed for the components of the CO_3_^2−^ band, corresponding to A and B-type of substitution (Fig. 5a,c), located both in the region 1550–1350 cm^−1^ and 890–860 cm^−1^, which corresponds to the different CO_3_^2−^ content in enamel apatite. The analysis of IR reflection spectra obtained from the enamel micro-areas of teeth with fluorosis shows that the profile of the spectral band of the radical PO_4_^3−^ in the enamel zones at different depths is practically indistinguishable (Fig. 6b). Note that the mode attributed to the B-type substitution of CO_3_^2−^ does not change, while the vibration mode intensity of CO_3_^2−^ ν_3_ attributed to A-type of substitution is significantly reduced in comparison with the similar mode for intact teeth (Fig. 6a,c).

It should be noted that the modes of CO_3_^2−^ at ν_3_ in the region 1540–1400.3 cm^−1^ and ν_1_ in 880–870 cm^−1^, corresponding to A and B-type substitution, are more pronounced than in Raman spectra for the same samples due to the bands from the radical PO_4_^3−^ overlapping with the modes of CO_3_^2−^. Therefore, it is possible to estimate the features of mineralization of enamel apatite occurring in fluorosis based on the ratio of the intensity of vibrational bands of CO_3_^2−^ A- and B-substitution. The average value of the ratio of the proportion of CO_3_^2−^ replacing phosphate or hydroxyl radicals (R [A-type/B-type]) in the enamel micro-areas near the surface (point 1) and about half of its thickness (point 2) is shown in Fig. 8. The results are presented in the form of mean ± standard deviation.

**Figure 7:**
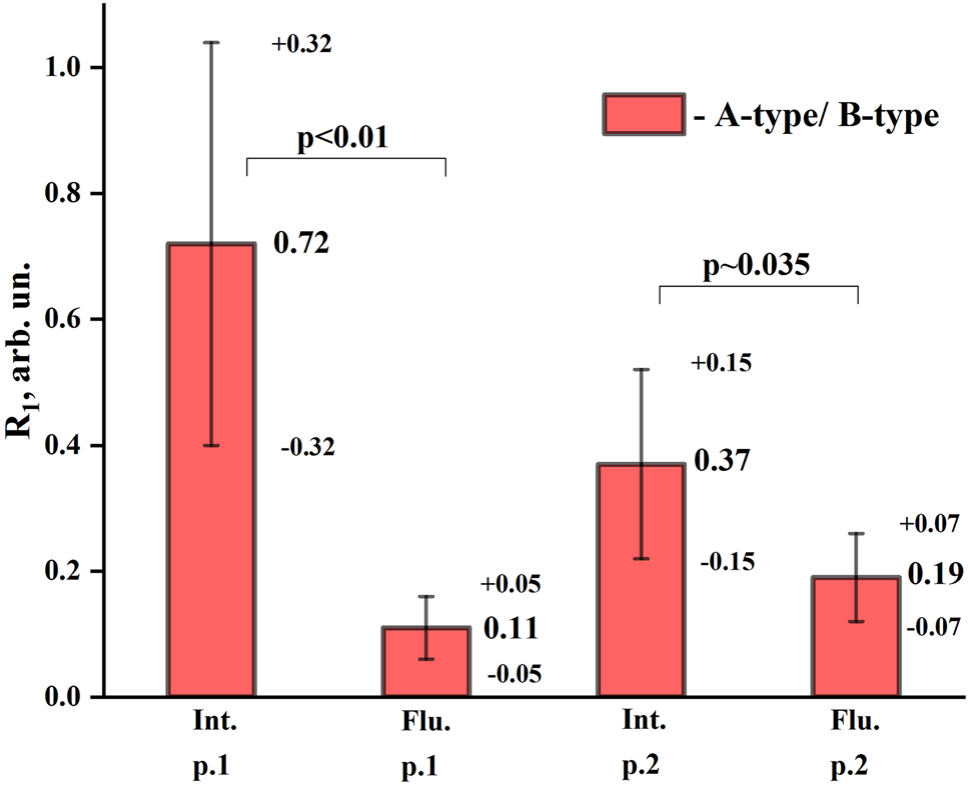
The ratio R [A-type/B-type] of the proportion of CO_3_^2−^ replacing phosphate or hydroxyl radicals (A and B-type of substitution) in the apatite of native dental hard tissue and the early stages of fluorosis from the surface (point 1) and deep (point 2) layers of enamel.

The obtained results show that the mean value of R [A-type/B-type] ratio in the fluorotic enamel in (experimental group of patients) is of about ~ 0.72, while for the intact enamel (control group of patients) the value of R [A-type/B-type] ratio is at the level of about ~ 0.11. Moreover, it can be clearly seen (Fig. 7), that for the intact enamel R [A-type/B-type] ratio is reduced from surface layers to the deep ones, while for the fluorotic enamel those changes are insignificant.

The statistical analysis of data using Mann–Whitney U-test showed statistically significant differences in the mean values of R [A-type/B-type] ratio between control and experimental groups, *p* < 0.01 for surface layers (point 1), and *p* ~ 0.035 for deep layers (point 2).

## Discussion

Previously, several studies by González-Solís et al.^15^ and Zavala-Alonso et al.^18^ showed that the chemical differentiation between native dental hard tissue and those affected by fluorosis may be associated with a difference in the intensity of the phosphate mode PO_4_^3−^ ν_1_ 960 cm^−1^ and carbonate ion mode CO_3_^2−^ ν_1_ 1073 cm^−1^ in Raman spectra. The intensity of these bands decreases as the degree of fluorosis increases, due to the large number of fluorine atoms introduced into the enamel apatite lattice increasing the level of phosphate mineralization. This, in turn, causes dissociation of hydroxyapatite and dissolution of carbonate ions in the presence of fluorine^15,18^. However, as it has been shown by González-Solís et al.^15^ and Zavala-Alonso et al.^18^, statistically significant changes in the intensities are only observed in cases of average and severe affection, not in the initial stage of pathology. Therefore, this method is ineffective to distinguish between healthy and fluorotic enamel with a TFI = 1–3. It should be noted that in severe cases of fluorosis, as shown by Gerth et al.^14^ and Campillo et al.^17^, the inclusion of fluorine in the OH- position resulted in a shift of the Raman scattering band ν_1_ PO_4_^3−^ to higher frequencies. Our experimental data show that in the spectra of fluorotic enamel samples (Fig. 1d), the shift in the main band relative to intact enamel samples is not visible, which indirectly confirms the low fluorine content in apatite.

Using Raman spectroscopy, the CaF_2_ content could be determined directly by detecting a band localised around 322 cm^−1^, however, the limits of detection and quantitative determination of fluoride lie above 3%^14,43^. In our experimental spectra, we observed a low-intensity band in the region of 280–311 cm^−1^ (Fig. 3a), which is referred to the vibrations of Ca_II_–OH hydroxyapatite and Ca_II_–F from fluorapatite (FAp)^42^. Although this band has a more pronounced shape and intensity in the spectra of fluorotic enamel, to use such an approach for screening of fluorosis with TFI ~ 1–3 is, in our opinion, is quite difficult.

It is known from literature that substitutions of different types in the crystal lattice of calcium hydroxyapatite are represented in the Raman spectra due to the emergence of additional active vibrational bands or the appearance of features in the form of a spectrum of stoichiometric HAP^53^. The formation of carbonate substituted hydroxyapatite is due to the anionic substitution of PO_4_^3−^ group in the apatite by CO_3_^2−^ (B-type substitution)^30,44,49,53^. This leads to a shift of the high-intensity mode ν_1_ PO_4_^3−^ into the low-frequency region, as well as the appearance of additional modes in the region of 1076 cm^−1^^44,47,49,50,53,56^. Less probable is the A-type substitution, where the OH group is replaced by the radical CO_3_^2−^. In Raman spectra, a low-intensity band in the region of 1105 cm^−1^ is shown^44,47,49,53^ to exist. From the analysis of the experimental spectral data (Fig. 1–3), it follows that both types of substitution (A and B-type) are present in intact and fluorotic enamel samples. In the case of enamel fluorosis, the A-type mode CO_3_^2−^ intensity is by several times lower in both surface and deep enamel layers (see Fig. 6), possibly due to the inclusion of fluorine atoms in the apatite structure^15,43,46,50^. It should be also noted that according to the results of Leroy et al.^46^ PO_4_^3−^ ν_2_ and PO_4_^3−^ ν_4_ modes in the Raman spectra of enamel were more broad than in the spectra of FAp. Since the Full Width at Half Maximum (FWHM) of the PO_4_^3−^ is selected as a crystallinity index^57^, then just the difference in crystallinity between ideally crystallized FAp and carbonate-substituted enamel apatite is the factor providing an increase of FWHM in the latter case.

However, based on the results obtained in our work, including the decomposition of PO_4_^3−^ ν_2_ and PO_4_^3−^ ν_4_ modes into the components (see Table 2), in case of enamel affected by fluorosis in the initial stages of disease (TFI ~ 1–3) an increase of FWHM of A, E_1_ and E_2_ bands is observed relative to those in the intact enamel. This fact can be attributed to the formation of the defects stipulated with the change of carbonization (mineralization) of the enamel apatite^44,45,47,49^.

The study of fluorotic enamel samples by infrared reflection spectroscopy using synchrotron radiation in different layers of enamel (Figs. 4–6) shows high stability of the ratio of intensities of the modes PO_4_^3−^ ν_3_ 1094, 1060, 1048, 1040 cm^−1^, PO_4_^3−^ ν_1_ 956.4 cm^−1^, which confirms the Raman spectroscopy data on the stability of the crystal lattice of apatite in fluorosis^37,42^. In this case, in the infrared spectra, similar to what is observed by Raman spectroscopy, a redistribution of the intensity of modes CO_3_ ν_3_ localised near 1540.7, 1446.6, 1401.3 cm^−1^ can be observed and it is associated with A and B-type substitution. This confirms that the A-type substitution defects in fluorotic enamel are reduced due to the inclusion of fluorine atoms in the apatite.

It should be noted that the intensity of the mode 1401.1 cm^−1^ and the shift of its position relative to the literature value of 1420 cm^−1^ (Figs. 5a and 6a) is due to the presence of a small amount of organic component in the enamel associated with CH group, the most intensive lines of which (symmetric CH_3_ bending, stretching of COO) arise in the region 1412–1396 cm^−1^, and overlap with the band of CO_3_^2−^ ν_3_^34,37,40^. However, the analysis of the low-intensity vibrational bands of CO_3_ ν_1_ in IR reflection spectra in the region of 890–860 cm^−1^, where (in contrast to the Raman spectra) there is no overlap with other vibrations (Table 1), showed a similar trend in the transformation of intensities for A and B-type carbonate substitutions in fluorotic enamel (Figs. 5c and 6c). The decrease in the proportion of CO_3_^2−^ substituting hydroxyl radical (A-type) relative to the B-type in the initial stage of fluorosis development, i.e. under the influence of a low concentration of fluorine, is most likely due to the proximity of the radii of ions and the equivalent substitution of F^−^ and OH^−^ making it easier for F^−^ to occupy the OH nodes in the apatite crystal, forcing most of the CO^2−^_3_ groups to get in the B node^53,56,58,59^, which ultimately affects the ratio of A and B in the apatite of fluorotic enamel. It is important to note that statistical analysis of the data shows significant differences in mean values of the ratio R [A-type/B-type] between the control and experimental groups, and for the surface layers, the level of significance is *p* < 0.01 (Fig. 7).

It is clearly seen that the R [A-type/B-type] ratio in the upper layers of fluorotic enamel is, in fact, by the order of magnitude less than in the intact enamel. Note, that in the deeper layers of fluorotic enamel located approximately in the middle of enamel layer this ratio is roughly twice lower than in the similar points of enamel layer in the intact teeth.

Thus, the use of Raman and IR-spectroscopy data regarding the mineralization of apatite enamel obtained in the work can distinguish between healthy and affected dental hard tissues in the initial stages of fluorosis, therefore they can be used to develop a highly sensitive approach for the screening of fluorine intoxication of enamel.

## Conclusion

In conclusion, the optical microspectroscopy analysis of enamel affected by fluorosis shows that in the initial stages of disease (TFI ~ 1–3), fluorine atoms are introduced into the enamel apatite, resulting in the formation of fluorinated substituted apatite. Moreover, micro-areas of fluorotic enamel contain defective hydroxyapatite, where hydroxyl groups are replaced by fluorine atoms with the displacement of CO^2−^_3_ from A-type defects in the apatite lattice. This is indicated by changes in the profile of the corresponding bands and redistribution of the intensity of Raman and IR reflection spectra components. Furthermore, the analysis of the spectral data shows that the inclusion of fluorine atoms in the initial stages of the disease does not lead to the elimination of carbonate anion from the B-type defects in the apatite enamel.

Mineralization of enamel in the presence of increased fluorine also leads to stabilization of the apatite structure, as evidenced by the stable ratio of intensities of modes PO_4_/CO_3_ (580–615 cm^−1^ and 1045–1080 cm^−1^) in the different enamel layers. The detected features can be used to develop a new diagnostic method for the early forms of fluorosis with a TFI ~ 1–3.

The use of a non-invasive diagnostic approach to monitor the increased fluoride content of hard tissues of human teeth will help to control fluorine intoxication of enamel, thereby providing an effective reduction of caries as well as reducing the probability of the disturbed development of teeth and possibly other organs and systems.

## Materials and methods

### Experiment design

Two groups of teeth samples from patients aged 18–25 by orthodontic indications were investigated, the first (control) group consisted of molars with intact enamel and the second (experimental) group contained molars with enamel affected by fluorosis. The teeth with increased fluoride content were collected from patients from a region of Russia, where there was increased fluoride content in drinking water ~ 1.5 ppm. The control group was collected in another region where the natural fluoride level in water was ~ 0.25 ppm.

**Figure 8:**
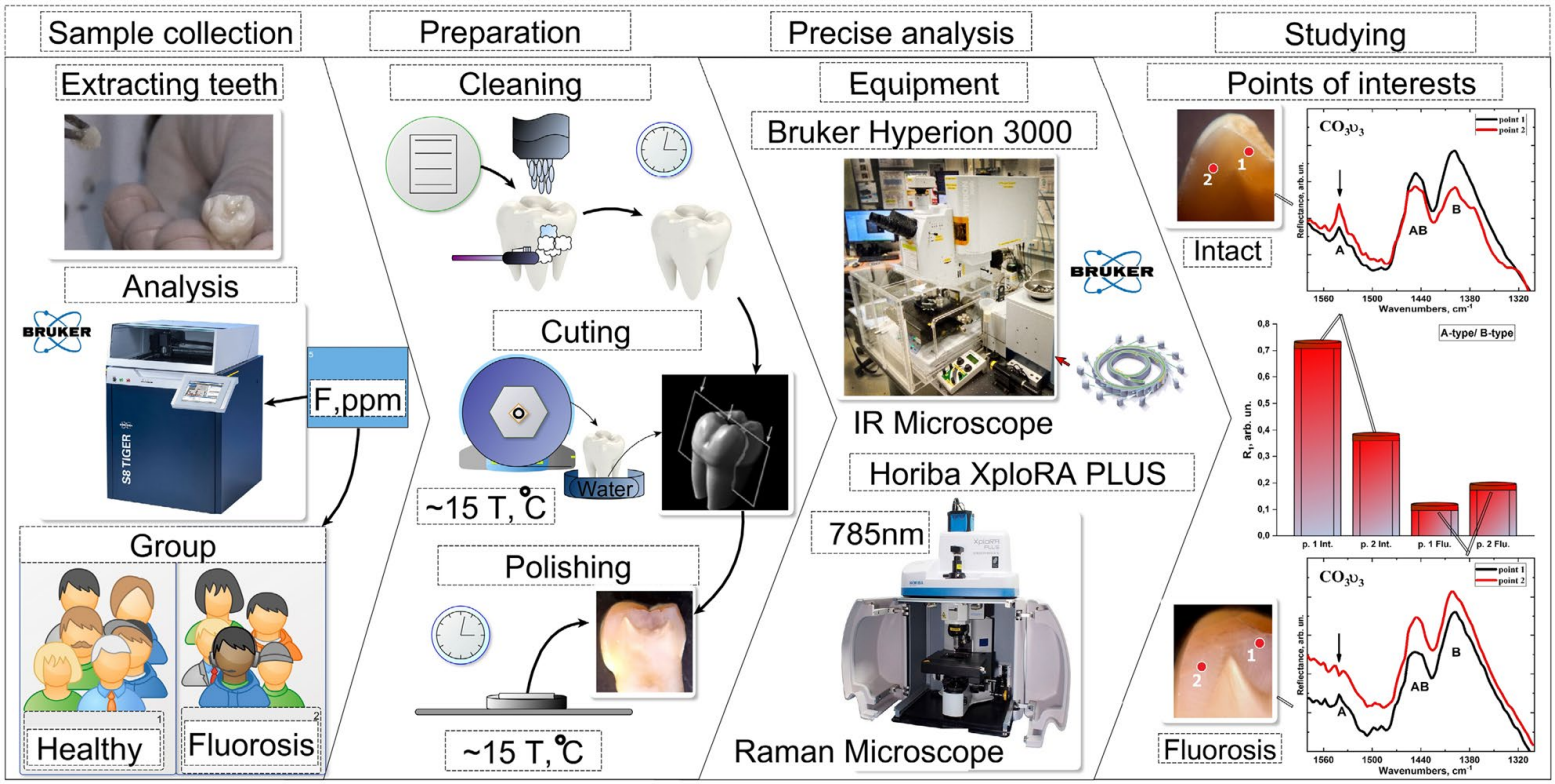
Design of experiment, preparation and study of samples using infrared and Raman microspectroscopy methods.

Patients were informed about the study and gave their written consent to participate before the teeth were extracted. Immediately after removal, the samples were cleaned and disinfected in an ultrasound bath. The teeth were washed in distilled water and dried, then stored frozen until further analysis. As shown previously^60^, freezing did not affect the properties of the tooth tissue. Schematically, the design of the experiment and all its stages are shown in Fig. 8.

### Ethics declarations

All participants provided their written consent for participation. The Ethics Committee of Voronezh State University affirmed the performed examination (number of permission 001.004-2020). The examination was made in accordance with the approved principles.

All experiments and data collections were performed in accordance with relevant guidelines and regulations, including that all human participants provided informed consent and data collection and handling followed the Helsinki declaration.

### Sampling technique

Initially, the composition of surface enamel layers was studied by wave dispersion X-ray fluorescence analysis using the S8 TIGER, Bruker spectrometer. Each sample was then analysed to determine the severity of fluorosis and classified according to the Thylstrup–Fejerskov scale (TFI) and previous data^60^. TFI links the clinical manifestation of fluorosis to the pathological changes occurring in enamel and is based on a 10-point scale, where zero is the state of the intact tooth tissue and 9 is the state of the most severely affected tooth tissue during fluorosis. At the same time, Vieira et al.^60^ compared the fluorine concentration in enamel layers to each level of the TFI scale.

The teeth samples in this study had a fluoride content in the enamel of ~ 200 ± 20 ppm, which according to Ref.^60^, corresponds to the early stage of fluorosis development (TFI = 1–3). In the control group of samples, molars in which surface enamel layers had fluorine content of ~ 100 ± 20 ppm were selected, which according to Ref.^60^, corresponds to the state of healthy enamel (TFI = 0). There were a total of 25 samples in each group.

Taking into account the requirements of microspectroscopic examination methods for sample geometry, plane-parallel segments of teeth were prepared as reported previously^20,27^. A special unit with a diamond disc and water cooling was used to separate the prepared teeth into segments (Fig. 8). For the teeth sections, the samples of intact teeth and those with fluorosis lesions were cut into slices and mounted on a thick polymer substrate using an epoxy adhesion. The top surface of the teeth slice was then polished using diamond paste.

### Equipment setup and sample scanning

#### Raman microspectroscopy

Raman scattering spectra were obtained using the confocal Raman microscope Xplora Plus, Horiba with spectral resolution: 1.5 cm^−1^ in the range: 200–2000 cm^−1^. Excitation was performed using a laser with a wavelength of 785 nm, power on the sample ~ 10–50 mW and the signal was collected using a 50× lens. In the micro-area selected for the study, the averaged spectra of 60 scans in the range of 200–2000 cm^−1^ were obtained. The size of the analysed micro-area was 20 µm^2^. Precision studies of tooth micro-areas were conducted in 100 µm steps from the surface to deep enamel layers. Spectral data processing, normalization, background correction, determination of line position and intensity, and also deconvolution of Raman modes by the components were performed using Origin 9.0 software.

#### IR microspectroscopy with the use of synchrotron radiation

The study was conducted on the Infrared Microspectroscopy (IRM) beamline (Australian Synchrotron, Victoria, Australia) using a Bruker Vertex 80v spectrometer coupled with a Hyperion 3000 FTIR microscope and a liquid nitrogen-cooled narrow-band mercury cadmium telluride (MCT) detector (Bruker Optik GmbH, Ettlingen, Germany). The synchrotron FTIR measurement of the tooth slices was performed in a reflectance mode, using the CsI window as an IR background reference. The spectral data acquisition was performed using an × 36 objective lens (NA = 0.50; Bruker Optik GmbH, Ettlingen, Germany), a beam focus of 6.9 µm in diameter and 128 co-added scans per spectrum. Background spectra were acquired on the surface of the CsI window, which was placed next to the teeth sample, using 256 co-added scans. All the synchrotron FTIR spectra were recorded within a spectral range of 3800‒700 cm^−1^ using 4-cm^−1^ spectral resolution. Blackman–Harris 3-Term apodization, Mertz phase correction, and zero-filling factor of 2 were set as default acquisition parameters using OPUS 7.2 software suite (Bruker Optik GmbH, Ettlingen, Germany). Spectral data processing (including rubberband-type background correction, peak detection and peak height determination) was performed using the procedures of OPUS 7.2 software.

### Statistical analysis

Statistical analysis was performed using a professional software package for statistical analysis SPSS versions. 19 for Windows, SPSS Inc., Chicago, Illinois, USA). Descriptive statistics in groups are given as average ± standard deviation.

## Data Availability

The data that support the findings of this study are available from the corresponding author upon a reasonable request.

## References

[1] Philip N, Suneja B, Walsh LJ (2018). Ecological approaches to dental caries prevention: Paradigm shift or shibboleth?. CRE.

[2] O’Mullane DM (2016). Fluoride and oral health. Commun. Dent. Health.

[3] Pitts NB (2017). Dental caries. Nat. Rev. Dis. Primers.

[4] Fewtrell L, Smith S, Kay D, Bartram J (2007). An attempt to estimate the global burden of disease due to fluoride in drinking water. J. Water Health.

[5] Carey CM (2014). Focus on fluorides: update on the use of fluoride for the prevention of dental caries. J. Evid. Based Dent. Pract..

[6] Hattab FN (2020). An update on fluorides and fluorosis with reference to oral health status in the gulf region: review. Asian J. Dent. Sci..

[7] DenBesten P, Li W (2011). Chronic Fluoride Toxicity: Dental Fluorosis. Monogr Oral Sci.

[8] Hicks J, Garcia-Godoy F, Flaitz C (2004). Biological factors in dental caries: role of remineralization and fluoride in the dynamic process of demineralization and remineralization (part 3). J. Clin. Pediatric Dent..

[9] Combes C, Cazalbou S, Rey C (2016). Apatite biominerals. Minerals.

[10] Aoba T (1994). Strategies for improving the assessment of dental fluorosis: focus on chemical and biochemical aspects. Adv. Dent. Res..

[11] ten Cate JM (1999). Current concepts on the theories of the mechanism of action of fluoride. Acta Odontol. Scand..

[12] Roveri N (2009). Surface enamel remineralization: biomimetic apatite nanocrystals and fluoride ions different effects. J. Nanomater..

[13] Philip N (2019). State of the art enamel remineralization systems: the next frontier in caries management. CRE.

[14] Gerth HUV, Dammaschke T, Schäfer E, Züchner H (2007). A three layer structure model of fluoridated enamel containing CaF_2_, Ca(OH)_2_ and FAp. Dent. Mater..

[15] González-Solís JL, Martínez-Cano E, Magaña-López Y (2015). Early detection of dental fluorosis using Raman spectroscopy and principal component analysis. Lasers Med. Sci..

[16] Tsuda H, Arends J (1993). Detection and quantification of calcium fluoride using micro-raman spectroscopy. Caries Res..

[17] Campillo M, Lacharmoise PD, Reparaz JS, Goñi AR, Valiente M (2010). On the assessment of hydroxyapatite fluoridation by means of Raman scattering. J. Chem. Phys..

[18] Zavala-Alonso V (2012). Analysis of the molecular structure of human enamel with fluorosis using micro-Raman spectroscopy. J. Oral Sci..

[19] Lee B-S, Chou P-H, Chen S-Y, Liao H-Y, Chang C-C (2015). Prevention of enamel demineralization with a novel fluoride strip: enamel surface composition and depth profile. Sci. Rep..

[20] Seredin P, Goloshchapov D, Prutskij T, Ippolitov Y (2015). Phase transformations in a human tooth tissue at the initial stage of caries. PLoS ONE.

[21] Buchwald T, Okulus Z, Szybowicz M (2017). Raman spectroscopy as a tool of early dental caries detection–new insights. J. Raman Spectrosc..

[22] Ko AC (2008). Early dental caries detection using a fibre-optic coupled polarization-resolved Raman spectroscopic system. Opt. Express.

[23] Choo-Smith L-P, Dong CCS, Cleghorn B, Hewko M (2008). Shedding new light on early caries detection. J. Can. Dent. Assoc..

[24] Muruppel AM, Coluzzi DJ, Parker SPA (2017). Laser-assisted diagnostics. Lasers in Dentistry—Current Concepts.

[25] Fried D, Wilder-Smith P, Ajdaharian J (2020). Optical methods for monitoring demineralization and caries. Oral Diagnosis.

[26] Pereira D (2018). Variation on molecular structure, crystallinity, and optical properties of dentin due to Nd:YAG laser and fluoride aimed at tooth erosion prevention. Int. J. Mol. Sci..

[27] Goloshchapov DL, Kashkarov VM, Ippolitov YA, Prutskij T, Seredin PV (2018). Early screening of dentin caries using the methods of Micro-Raman and laser-induced fluorescence spectroscopy. Results Phys..

[28] Slimani A (2017). Confocal Raman mapping of collagen cross-link and crystallinity of human dentin–enamel junction. J. Biomed. Opt..

[29] Buchwald T, Buchwald Z (2019). Assessment of the Raman spectroscopy effectiveness in determining the early changes in human enamel caused by artificial caries. Analyst.

[30] Fleet ME (2017). Infrared spectra of carbonate apatites: evidence for a connection between bone mineral and body fluids. Am. Mineral..

[31] Seredin P, Goloshchapov D, Ippolitov Y, Vongsvivut J (2019). Spectroscopic signature of the pathological processes of carious dentine based on FTIR investigations of the oral biological fluids. Biomed. Opt. Express BOE.

[32] Almhöjd US, Norén JG, Arvidsson A, Nilsson Å, Lingström P (2014). Analysis of carious dentine using FTIR and ToF-SIMS. Oral Health Dent. Manag..

[33] Simon JC (2016). Near-infrared imaging of secondary caries lesions around composite restorations at wavelengths from 1300–1700-nm. Dent. Mater..

[34] Jegova G, Titorenkova R, Rashkova M, Mihailova B (2013). Raman and IR reflection micro-spectroscopic study of Er:YAG laser treated permanent and deciduous human teeth. J. Raman Spectrosc..

[35] Desoutter A (2019). Cross striation in human permanent and deciduous enamel measured with confocal Raman microscopy. J. Raman Spectrosc..

[36] Seredin PV (2020). Organic–mineral interaction between biomimetic materials and hard dental tissues. Sovrem. Tehnol. Med..

[37] Porto IM (2010). Organic and inorganic content of fluorotic rat incisors measured by FTIR spectroscopy. Spectrochim. Acta Part A Mol. Biomol. Spectrosc..

[38] Chan KLA, Kazarian SG (2003). New opportunities in micro- and macro-attenuated total reflection infrared spectroscopic imaging: spatial resolution and sampling versatility. Appl. Spectrosc..

[39] Vongsvivut J (2019). Synchrotron macro ATR-FTIR microspectroscopy for high-resolution chemical mapping of single cells. Analyst.

[40] Lopes CCA, Limirio PHJO, Novais VR, Dechichi P (2018). Fourier transform infrared spectroscopy (FTIR) application chemical characterization of enamel, dentin and bone. Appl. Spectrosc. Rev..

[41] Nikolishin AK, Kislovskiĭ LD (1991). Infrared spectroscopy of the enamel in dental fluorosis. Stomatol. Mosk.

[42] Penel G (1997). Infrared and Raman microspectrometry study of fluor–fluor–hydroxy and hydroxy-apatite powders. J. Mater. Sci. Mater. Med..

[43] Tsuda H, Arends J (1997). Raman spectroscopy in dental research: a short review of recent studies. Adv. Dent. Res..

[44] Penel G, Leroy G, Rey C, Bres E (1998). MicroRaman spectral study of the PO_4_ and CO_3_ vibrational modes in synthetic and biological apatites. Calcif. Tissue Int..

[45] Salehi H (2012). Functional mapping of human sound and carious enamel and dentin with Raman spectroscopy. J. Biophoton..

[46] Leroy G, Penel G, Leroy N, Brès E (2002). human tooth enamel: a Raman polarized approach. Appl. Spectrosc..

[47] Ko AC-T (2005). Ex vivo detection and characterization of early dental caries by optical coherence tomography and Raman spectroscopy. J. Biomed. Opt..

[48] Torres CP (2018). FT-Raman spectroscopy, µ-EDXRF spectrometry, and microhardness analysis of the dentin of primary and permanent teeth. Microsc. Res. Tech..

[49] Spizzirri PG, Cochrane NJ, Prawer S, Reynolds EC (2012). A comparative study of carbonate determination in human teeth using Raman spectroscopy. Caries Res..

[50] Pessanha S (2020). Evaluation of the effect of fluorinated tooth bleaching products using polarized Raman microscopy and particle induced gamma-ray emission. Spectrochim. Acta Part A Mol. Biomol. Spectrosc..

[51] Anwar Alebrahim M, Krafft C, Sekhaneh W, Sigusch B, Popp J (2014). ATR-FTIR and Raman spectroscopy of primary and permanent teeth. Biomed. Spectrosc. Imaging.

[52] Penel G (2003). Raman microspectrometry studies of calcified tissues and related biomaterials. Dent. Med. Probl..

[53] Khan AF (2013). Raman spectroscopy of natural bone and synthetic apatites. Appl. Spectrosc. Rev..

[54] Mihály J, Gombás V, Afishah A, Mink J (2009). FT-Raman investigation of human dental enamel surfaces. J. Raman Spectrosc..

[55] Bachmann L, Diebolder R, Hibst R, Zezell DM (2003). Infrared absorption bands of enamel and dentin tissues from human and bovine teeth. Appl. Spectrosc. Rev..

[56] Chen J (2014). Effects of fluorine on the structure of fluorohydroxyapatite: a study by XRD, solid-state NMR and Raman spectroscopy. J. Mater. Chem. B.

[57] Dauphin Y (2020). Using microstructures and composition to decipher the alterations of rodent teeth in modern regurgitation pellets—a good news-bad news story. Minerals.

[58] Zhu Q-X, Li Y-M, Han D (2015). Co-substitution of carbonate and fluoride in hydroxyapatite: effect on substitution type and content. Front. Mater. Sci..

[59] Fathi MH, Zahrani EM (2009). Fabrication and characterization of fluoridated hydroxyapatite nanopowders via mechanical alloying. J. Alloys Compd..

[60] Vieira APGF, Hancock R, Limeback H, Maia R, Grynpas MD (2004). Is fluoride concentration in dentin and enamel a good indicator of dental fluorosis?. J. Dent. Res..

